# Mass Mortality in Terns and Gulls Associated with Highly Pathogenic Avian Influenza Viruses in Caspian Sea, Kazakhstan

**DOI:** 10.3390/v16111661

**Published:** 2024-10-24

**Authors:** Aidyn Kydyrmanov, Kobey Karamendin, Yermukhammet Kassymbekov, Klara Daulbayeva, Temirlan Sabyrzhan, Yelizaveta Khan, Sardor Nuralibekov, Barshagul Baikara, Sasan Fereidouni

**Affiliations:** 1 Research and Production Center for Microbiology and Virology, Almaty A26T6C0, Kazakhstan; 2 Research Institute of Wildlife Ecology, University of Veterinary Medicine Vienna, 1160 Vienna, Austria

**Keywords:** highly pathogenic influenza viruses, Caspian Sea, Caspian tern, Caspian gull, H5N1 subtype, Kazakhstan, outbreak

## Abstract

Mass mortality in Caspian terns (*Hydroprogne caspia*), Pallas’s gulls (*Ichthyaetus ichthyaetus*), and Caspian gulls (*Larus cachinnans*) was recorded on the northeastern shores of the Caspian Sea in June 2022. More than 5000 gulls and terns died due to the outbreak. The outbreak was investigated in the field, and representative numbers of samples were collected and analyzed using pathological, virological, and molecular methods. Highly pathogenic avian influenza A (H5N1) viruses were detected and isolated from samples collected from dead birds. Genetic and phylogenetic analyses indicated that the hemagglutinin (*HA*) genes belonged to the clade 2.3.4.4.b of the H5Nx HPAI viruses, B2 sub-lineage, and were closely related to the highly pathogenic influenza viruses, caused an outbreak in wild birds with a high mortality rate in the western part of the Caspian Sea.

## 1. Introduction

Avian influenza infections are caused by influenza A viruses (IAVs) in a diverse range of wild and domestic birds. Four types of influenza viruses are currently recognized, designated A through D. It is notable that types B, C, and D primarily affect mammals and have not been observed in birds. However, IAVs have a wider range of hosts, including birds and various mammalian taxa such as equids, felids, canids, pinnipeds, suids, and humans [1]. Despite the diverse range of the hosts, wild birds are regarded as the primary natural reservoir of IAVs [2]. To date, 19 hemagglutinin (*HA*) subtypes and 11 neuraminidase (*NA*) subtypes have been described [3]. With the exception of the two bat-specific *HA* subtypes (H17N10 and H18N11), all other subtypes have been identified in wild birds. Since the emergence of the highly pathogenic avian influenza (HPAI) viruses H5 A/Goose/Guangdong/1/1996 (GsGd) lineage in 1996, there have been extensive global infections and outbreaks with high mortality rates in numerous wild bird species [4,5]. This constituted a novel development in the ecology and epidemiology of AIVs, as wild birds had not previously been considered as potential victims or carriers of highly pathogenic avian influenza viruses (HPAIVs) [1]. It has been postulated that the HPAI H5 GsGd lineage and its progeny could disseminate and spread globally most likely via infected migratory birds as well as infected domestic poultry, causing devastating outbreaks in domestic and wild birds worldwide [4,5,6]. In Europe, the HPAI H5 2.3.4.4. clade has been the predominant lineage since 2014, and a novel lineage of this clade emerged in 2020 [7]. This resulted in the largest global HPAI outbreak to date, with an increasing number of reports indicating the spillover of the virus to mammalian species [8]. The global dissemination of HPAI H5N1 viruses belonging to the clade 2.3.4.4b has resulted in the infection of rare and highly protected avian species with these viruses, with significant consequences for the affected populations. A number of high mortality outbreaks in colony-breeding seabirds were reported globally during the 2021–2022 period [8,9], raising great concern about the further spread of infection to immunologically naive populations of vulnerable species. In July 2021, the HPAIV subtype H5N1 clade 2.3.4.4b caused die-offs of Great Skua breeding populations on several Scottish islands [9]. Furthermore, the virus severely affected the majority of Northern gannets (*Morus bassanus*) colonies in Iceland, the Scottish islands, Ireland, Canada, Germany, and Norway between April and September 2022, significantly reducing their breeding success and the number of nesting colonies [10]. It is estimated that approximately 1500 vulnerable Caspian terns (*Hydroprogne caspia*) perished as a result of infection with H5N1 HPAIVs on Lake Michigan in the USA in June 2022 [11,12,13]. Furthermore, during almost the same period, the virus caused the deaths of over 8000 adult sandwich terns (*Thalasseus sandvicensis*) and thousands of their chicks in the Netherlands and Germany [14,15], more than 800 black-headed gulls (*Chroicocephalus ridibundus*) and smaller numbers of sandwich terns and Mediterranean gulls (*Ichthyaetus melanocephalus*) in three colonies in France [16]. In 2013, a high mortality outbreak in Caspian terns was reported on the Zuid-West Island in the northern Caspian Sea due to an adenovirus infection [17]. Here, we report the mass mortality of Caspian terns and Caspian gulls breeding colonies in the northern Caspian Sea due to the HPAIV subtype H5N1 clade 2.3.4.4b. A genetic and phylogenetic analysis of the isolate A/Caspian tern/Atyrau/9184/2022(H5N1), indicated that the *HA* gene belonged to the clade 2.3.4.4.b of the H5Nx HPAI viruses, B2 sub-lineage. Nevertheless, the *HA*, *NA* and other six genes of the virus exhibited highest degree of genetic relatedness to the HPAIVs of clade 2.3.4.4.b, which were isolated from a wild bird outbreak in Astrakhan (Russia) in 2022.

## 2. Materials and Methods

### 2.1. Field Study

**Figure 1:**
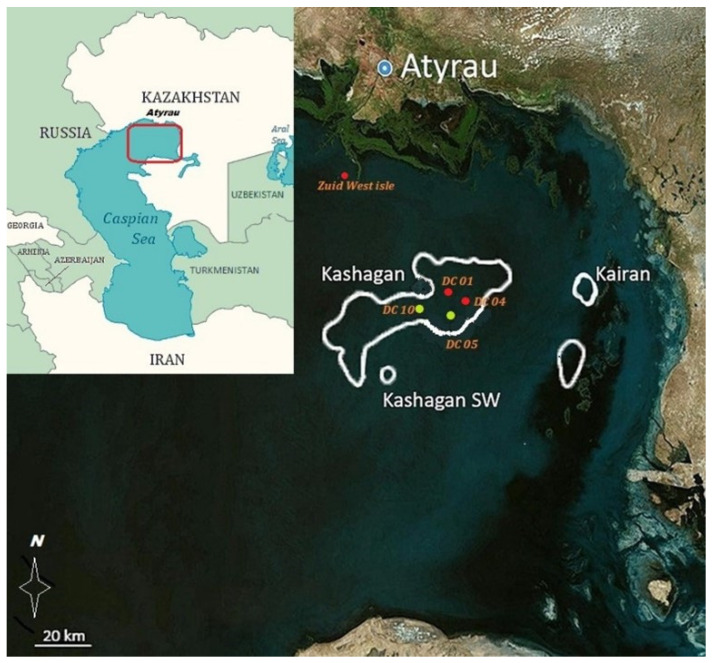
Satellite map of the northern Caspian Sea illustrates the main die-off sites. The red dots indicate the locations where bird die-offs were registered, and the lime-colored dots represent the isles where avian colonies have remained uninfected (the satellite map was modified after A. Kenzhegaliev et al. [19]).

A mass mortality of Caspian terns (*Hydroprogne caspia*) and sympatric Caspian gulls (*Larus cachinnans*) nesting on artificial islands (designated as DC 01 and DC 04) in the Kashagan offshore field (46°10′ N 51°35′ E) and of Zuid-West Island (part of the State Nature Reserve “Akzhayk”) in the northern part of the Caspian Sea was reported on 19 June 2022 (Figure 1). Wildlife mortality in the vicinity of oil fields in the North Caspian Sea is a matter of special attention of environmental protection authorities and the general public. Accordingly, an increase in the number of dead and sick birds in the colonies is a cause for concern for specialists in offshore monitoring stations and wildlife rangers to report the fatalities to veterinary authorities. The outbreak persisted until 4 July 2022 [18]. The number of dead birds of each species was counted one week after the first carcass removal, and all data were collected in a database after each collection and monitoring visit until the last moribund or sick bird was observed. Sick and dying birds were abandoned on their nesting grounds and were not included in the statistical analysis.

The preliminary diagnosis of HPAI H5N1 was made by the National Veterinary Reference Center (Astana) [18], and therefore, all dead birds were considered potentially infected with the virus. Consequently, special biosafety considerations were implemented in the outbreak area. In accordance with the relevant health and safety regulations, all personnel involved in the collection of carcasses were provided with the necessary personal protective equipment (gloves, safety goggles, shoes, N95-type masks, hard hats, and Tyvek® full-body suits). The carcasses were collected in heavy-duty, leak-proof disposable plastic bags for subsequent transportation by ship to the designated incineration site. Subsequently, the stacked bags of bird carcasses were incinerated at the Atyrau Municipal Veterinary Station.

Data collection on the HPAI H5N1 outbreak in seabird colonies, including collection of records and registration, the gathering of colony-specific environmental data, the documentation of outbreak control measures, and the monitoring of carcass disposal process, was managed and conducted by the Kazakhstan Agency of Applied Ecology (KAAE) for the North Caspian Operating Company (NCOC) within the framework of the Wildlife Sustainability Survey Program for this region.

### 2.2. Sample Preparation and Nucleic Acid Extraction

The postmortem examination of the dead birds was performed by a wildlife specialist veterinarian in the field, considering biosafety regulations. Thirty-four tracheal and cloacal swabs and 20 tissue samples (brain and internal organs) from 17 dead birds (7 Caspian terns, 5 Caspian gulls, and 5 Pallas’s gulls), were collected in cryovials containing virus transport media. The samples were stored in a cryogenic liquid nitrogen container (−196 °C) and transferred to a biosafety level 2 (BSL2) laboratory at the Research and Production Center for Microbiology and Virology, Almaty, Kazakhstan. RNA was extracted from the swabs and tissue samples using the QIAamp Viral RNA kit (Qiagen, Hilden, Germany).

### 2.3. Diagnostic Procedure

Samples were tested by influenza A virus multiplex reverse transcription-PCR (RT-PCR) using matrix (*M*), H5, and/or N1 gene-specific primers [20]. In addition, samples were tested for adenoviruses and paramyxoviruses (avulaviruses) to exclude other potential pathogens involved in the outbreak [8].

RT-PCR assays were performed using one-step protocols with the Qiagen RT-PCR Kit according to the manufacturer’s instructions. Virus isolation was performed for all samples in the BSL3 facilities of the Kazakh Scientific Research Veterinary Institute (Almaty, Kazakhstan), using specific pathogen-free (SPF) embryonated chicken eggs according to standard procedures.

### 2.4. Sequencing and Phylogenetic Analysis

Next-generation sequencing (NGS) was performed to complete genome sequences. For this purpose, viral RNA was extracted from allantoic fluid and subjected to reverse transcription (RT) and double-stranded DNA construction utilizing a QIASeq Stranded RNA Library Kit (Qiagen, Hilden, Germany) using random hexamer primer at a concentration of 100 pmol. Furthermore, the genomic libraries were generated using the NEBNext Ultra DNA Library Prep Kit for Illumina (New England Biolabs, Ipswich, MA, USA). The DNA fragmentation was carried out with a size selection of approximately 400–450 bp by AMPure^®^XP beads (Beckman Coulter Inc. Brea, CA, USA) according to the manufacturer’s protocol. Paired-end sequencing was conducted on the MiSeq Illumina platform using the MiSeq Reagent v.3 kit (600 cycles) (Illumina, San Diego, CA, USA). The sequence data obtained were trimmed at the 3′ and 5′ ends with an error probability limit of 0.05, and de nova assembly was performed using MEGAHIT v1.2.9, with default parameters [21]. Phylogenetic trees were constructed using a maximum likelihood approach (MEGA 11.0 software) based on a GTR + I + G model. The phylogenetic trees were midpoint-rooted. The numbers at the nodes indicate the maximum likelihood bootstrap values of 500 replicates under the specified model. Only values exceeding 70 are presented.

## 3. Results

### 3.1. Pathological Findings

**Figure 2:**
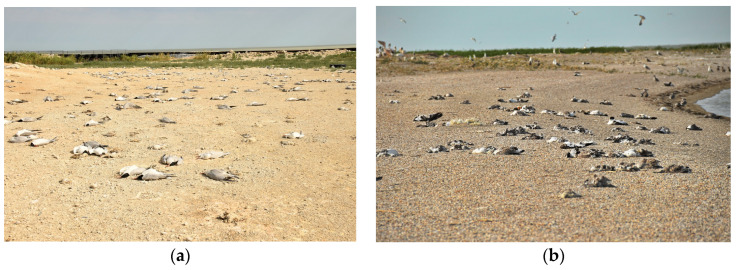
Mortality among the tern and gull populations on the DC-04 (**a**) and Zuid-West (**b**) islands.

Over 2200 dead adults, fledglings, and downy chicks of Caspian terns, and 550 adults and feathered chicks of Caspian gulls were counted on DC 01 and DC 04 isles. In addition, hundreds of weak and moribund individuals were observed among adult and juvenile Caspian terns on these islands. In contrast, no mortality was observed in other species, such as Little terns (*Sterna albifrons*), Pallas’s gulls (*Ichthyaetus ichthyaetus*), Great egrets (*Egretta alba*), and Grey herons (*Ardea cinerea*), nesting in close proximity on the islands. Furthermore, in a mixed population of Caspian terns and gulls (approximately 3000 birds) and Pallas’s gulls (approximately 5000 birds) breeding on Zuid-West Island, carcasses of approximately 1000 Caspian terns, 1200 Pallas’s gulls, and 300 Caspian gulls were counted (Figure 2, Table 1). It is noteworthy that despite the presence of the same gull and tern species on artificial islands DC 05 and DC 10, no bird mortality was observed.

**Figure 3:**
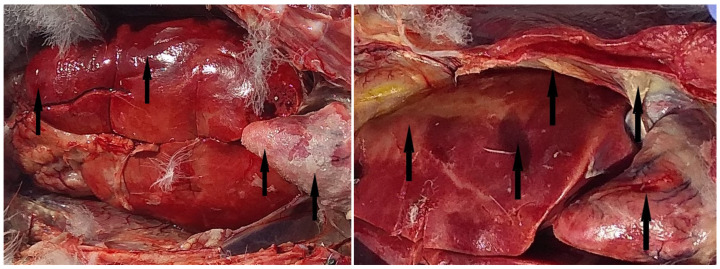
The livers, hearts, and air sacs of two dead Caspian gulls exhibited evidence of hepatitis, hepatic hemorrhage, pericarditis, and airsacculitis, respectively. The black arrows indicate the presence of pathological lesions on the organs.

The clinical signs observed in the sick birds included a high respiratory rate and neurological signs such as wing paresis, an inability to fly, torticollis, and opisthotonos. The post-mortem findings in the field included subcutaneous hemorrhages, intestinal hemorrhages, hepatitis, hepatic hemorrhages, pericarditis in conjunction with airsacculitis (Figure 3), and cerebral congestion. In some individuals, the peri-cloacal area was contaminated with fecal matter as a sign of diarrhea.

### 3.2. Molecular Diagnostic Findings

All 34 tracheal and cloacal swabs and 20 tissue samples tested positive by one of influenza A virus multiplex RT-PCR tests designed for the *M*, *H5*, and *N1* genes; however, they were negative for adenoviruses and paramyxoviruses.

Virus isolation in SPF embryonated chicken eggs yielded the influenza A virus isolate, designated as A/Caspian tern/Atyrau/9184/2022(H5N1). Virus isolation for other samples did not yield any hemagglutinating virus, which is likely attributable to the conditions under which the samples were transported. A complete genome comprising a 13,150 nucleotide sequence was recovered from 2,260,410 total NGS reads using the de Nova assembly (accession numbers: OQ804408–OQ804415). Based on the amino acid sequence of the *HA* proteolytic cleavage site (PLREKRRKR*GLF), the isolate was classified as HPAIV.

### 3.3. Phylogenetic Analysis

**Figure 4:**
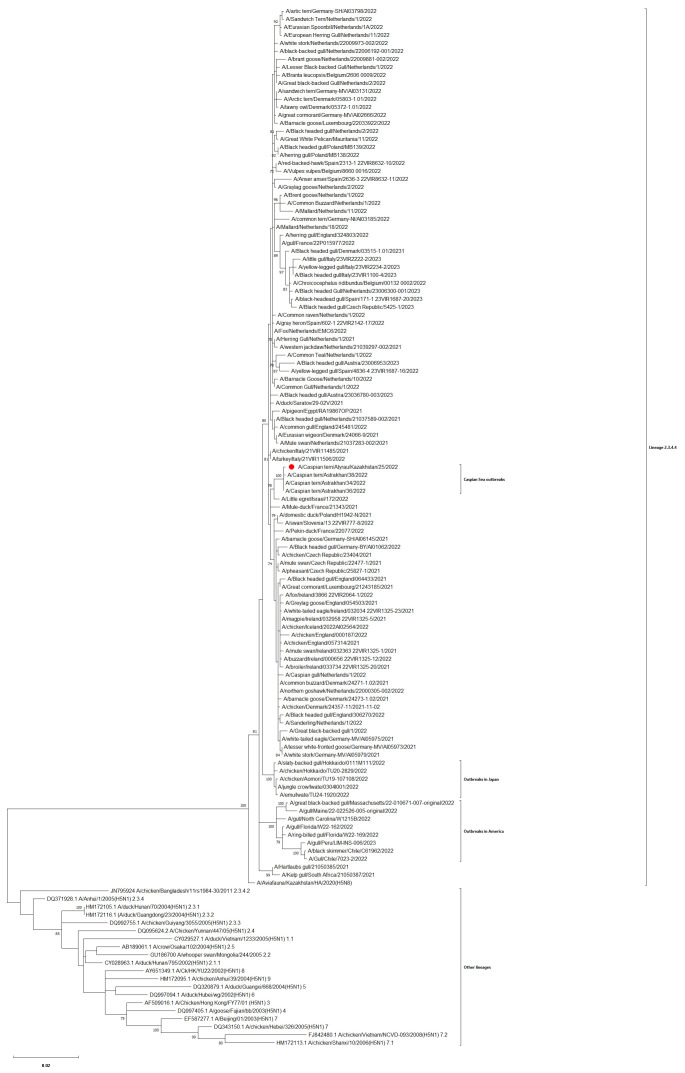
The phylogenetic tree of the full-length coding sequence of the *HA* gene of the HPAIV H5N1 subtype A/Caspian tern/Atyrau/9184/2022 (marked with red dot) and other related viruses isolated mainly in 2021, 2022, and 2023 from wild birds (GenBank and GISAID databases). The numbers at the nodes indicate the maximum likelihood bootstrap values of 500 replicates under the specified model. Only bootstrap values >70 are depicted. The bar represents a substitution rate of 0.02 nucleotides per site.

The phylogenetic analysis indicated that the *HA* genes belonged to the clade 2.3.4.4.b of the H5Nx HPAI viruses, B2 sub-lineage (Figure 4), with isoleucine as the sub-lineage genetic signature at HA position 548 instead of methionine for the B1 sub-lineage [22].

The HA, NA, and other genes of the Caspian tern were closely related to were closely related to the Russian isolates of clade 2.3.4.4.b from 2022 and other viruses from this clade which infected wild birds in Europe, Asia, and Africa in the same period (Figures S1–S7).

## 4. Discussion

In summary, the HPAIV outbreak in the Northern Caspian Sea occurred concurrently with similar outbreaks in sandwich terns in the Netherlands and Germany [14,15], black-headed gulls and sandwich terns in France [16], and Northern Gannets in multiple European countries [10]. A notable mortality rate among wild migratory waterbirds was observed on Maliy Zhemchuzhniy Island in the Russian part of the Caspian Sea, attributed to clade 2.3.4.4.b of H5N1 HPAIV [23], and our phylogenetic analysis also indicated a close relatedness among the viruses associated with the outbreaks in the western and eastern sides of the Caspian Sea. Gulls and terns serve as hosts for influenza A viruses of subtypes H13 and H16, and they harbor gull-specific lineages of *NP* and *M* genes of these AIV subtypes. At the time of writing, seabirds continued to be affected by the HPAI H5N1 clade 2.3.4.4b genotype EA-2022-BB, albeit at a lower rate than in previous epidemiological years [24].

The virus caused high mortality rates among adult birds in all affected countries, leading to a reduction in the number of breeding colonies. Additionally, the impact on chicks also have been significant. While adult birds died mainly due to the systemic infection, the mortality of chicks may have been at least partly was caused by starvation, as feeding was stopped because of the parents’ sickness and death [14]. 

Based on current report, the outbreak caused high adult and chick mortality in colonies of Caspian gulls and terns on both artificial islands and Zuid-West Island. However, carcasses of Pallas’s gulls were only found on Zuid-West Island. No reasonable hypothesis could be formulated to explain these differences. Nevertheless, comparable observations have been reported from the North Sea coasts where certain tern colonies remained unaffected [15].

The monitoring of the seabirds nesting colonies on the artificial islands (designated as DC isles) within the offshore complex at the Kashagan oil field has been scheduled as a daily task under the purview of the Industrial Environment Control of North Caspian Operating Company (NCOC). The program encompasses a daily observation of birds at offshore monitoring stations, seasonal specialized aerial bird surveys, and surface studies. In 2008, the monitoring system and surveys were expanded for all seasons. Furthermore, aero-visual surveys were conducted during the spring and autumn migration periods, and an additional autumn overflight survey was conducted. Accordingly, two autumn surveys, each spanning a duration of 4–6 weeks, have been scheduled for each year to cover extended autumn migrations for a diverse range of avian species. Furthermore, an additional aero-visual survey is conducted in mid-June to identify existing colonies and mass concentrations of birds that do not breed during this season. All results are recorded with references to coordinates. Since 2012, regular (seasonal) bird observations have been conducted from the vessels involved in offshore environmental surveys (scientific research vessels—SRVs) [25], thus enabling us for the timely identification of seabird mortality.

The Caspian tern HPAIV *NP* gene showed high genetic relatedness with a diverse group of Eurasian low and highly pathogenic avian influenza viruses, isolated from waterfowl and gulls. The *M* gene demonstrated similarities with diverse host origin H5N1 strains isolated mainly in Russia, Japan, and partially in Bangladesh and Egypt during 2021–2022 period. The analysis of the protein sequence of the *NP* and *M* genes of the Caspian tern HPAIV did not reveal any distinctive substitution or significant mutation.

The *NS1* gene of A/Caspian tern/Atyrau/9184/2022(H5N1) together with its closest related viruses of Astrakhan outbreak in 2022, exhibit a close phylogenetic relatedness to non-H5N1 Eurasian viruses, and it is therefore probable that the ancestor of these viruses was generated via reassortment with LPAIVs. These viruses have three previously undescribed, unique mutations/substitutions of Thr80Ile, Asp173Asn, and Pro212Ser in the NS1 protein. The Leu27Met-specific substitution in the NS1 protein of the Caspian tern virus has also been identified in other HPAIVs, particularly those that infected gulls in numerous European countries, including Austria, the Czech Republic, Italy, Belgium, and Spain. Notably, this substitution has not been identified in the previous outbreaks. Furthermore, other gene segments were also found to be closely related to the viruses isolated in Astrakhan in 2022 and other HPAIVs isolated from the birds of Laridae and/or Anatidae families during the outbreaks of 2020–2023. The isolate *PB1* and *PA* genes of the A/Caspian tern/Atyrau/9184/2022 demonstrated a high degree of genetic relatedness to isolates from Southern Asia, in addition to the isolates from the Astrakhan outbreak in 2022. Nevertheless, the *PB2* gene of this isolate, additionally showed a close relationship with the viruses from Eastern Europe, isolated from wild birds. Amino acid mutations, such as L13P in PB1 and N615K, were identified in PA gene of this virus that enhance the virulence of the H5N1 viruses in mammals (Table 2, [26]). The D619N substitution in the *PB1* protein of avian influenza viruses has been demonstrated to be associated with an increase in polymerase activity in human cells (Table 2, [27]). Moreover, the I61T mutation in the PA polypeptide may confer advantages for growth and adaptation of AIVs in humans (Table 2, ref. [28]). In contrast, the K237E substitution has been observed to increase polymerase activity to a modest extent [29]. These mutations illustrate the zoonotic potential of the A/Caspian tern/Atyrau/9184/2022 strain, and the impact of distinctive amino acid substitutions at the PB2 (A623T and S629C) and PB1 (R465G) polypeptides on the virus properties necessitates further investigation.

There is a global lack of regulations and action plans for prophylactic measures against HPAIV infection in seabird breeding colonies. Given that seabirds have a low annual reproductive output [14]; therefore, high adult and chick mortality will have a significant impact on population size. The North Caspian region represents a convergence point for the Black Sea–Mediterranean and East African–West Asian migratory flyways, and it is considered as a prospective epicenter for the emergence of novel and rare influenza A virus subtypes [3,30]. In early 2023, thousands of seabirds were severely affected by HPAIVs along the coasts of West African countries, including Senegal, Gambia, and Guinea-Bissau, with the main casualties in the birds of Laridae family including the Caspian tern, Royal tern (*Thalasseus maximus*), and grey-headed gull (*Larus cirrocephalus*) [31]. Although GPS tracking data of Caspian terns breeding in Northern Europe indicates a typical migration to the wintering grounds of Western Africa [32], a potential connection via migratory routes between the two outbreaks was considered. However, no sequencing data from those outbreaks was available in the GenBank and GISAID databases. It is recommended that intensive monitoring of wild bird populations, particularly Caspian gulls and terns on the Northern Caspian Sea Islands, be continued. This will provide an opportunity to evaluate the long-term effects of HPAIVs on those populations and the probability of HPAIV endemicity.

## Figures and Tables

**Table 1:** Population size and mortality rate of terns and gulls in affected colonies in Kazakhstan in 2022.

Affected Bird Species	Estimated Population on Island (2022)		Number of Found Dead Birds in a Colony		Percentage (%) of Found Dead Birds in the Colony		Mortality Rate (per 1000 Individuals)	
	D1 and D4	Zuid-West	D1 and D4	Zuid-West	D1 and D4	Zuid-West	D1 and D4	Zuid-West
Caspian tern	2400	2500	2198	1000	91.6	40.0	916	400
Caspian gull	600	500	530	300	88.3	60.0	883	600
Pallas’s gull	700	5000	27	1200	3.9	24.0	39	240

**Table 2:** Molecular markers associated with the adaptation of H5 AIVs to mammalian hosts.

Protein	H5 AIVs			Atyrau/9184/22	Phenotype
	Residue (AA)	Avian-Like Motif	Mammalian-Like Motif		
PB1	13	L	P	P	Enhanced virulence in mammalian species [26]
	619	A	T	A	Increased polymerase activity in human cells [27]
PA	61	I	T	I	Playing a vital role in the adaptation new host species [28]
	237	K	E	E	Increased polymerase activity [29]
	615	N	K/R	K	Enhanced virulence in mammalian species [26]

## Data Availability

Sequence data were submitted to GenBank. The accession numbers are provided in the manuscript.

## Supplementary Materials

The following supporting information can be downloaded at: https://www.mdpi.com/article/10.3390/v16111661/s1. Figure S1: Phylogenetic tree of the full-length coding sequence of the *PB2* gene of the HPAIV H5N1 subtype A/Caspian tern/Atyrau/9184/2022; Figure S2: Phylogenetic tree of the full-length coding sequence of the *PB1* gene of the HPAIV H5N1 subtype A/Caspian tern/Atyrau/9184/2022; Figure S3: Phylogenetic tree of the full-length coding sequence of the *PB2* gene of the HPAIV H5N1 subtype A/Caspian tern/Atyrau/9184/2022; Figure S4: Phylogenetic tree of the full-length coding sequence of the *NP* gene of the HPAIV H5N1 subtype A/Caspian tern/Atyrau/9184/2022; Figure S5: Phylogenetic tree of the full-length coding sequence of the *NA* gene of the HPAIV H5N1 subtype A/Caspian tern/Atyrau/9184/2022; Figure S6: Phylogenetic tree of the full-length coding sequence of the *M* gene of the HPAIV H5N1 subtype A/Caspian tern/Atyrau/9184/2022; Figure S7: Phylogenetic tree of the full-length coding sequence of the *NS* gene of the HPAIV H5N1 subtype A/Caspian tern/Atyrau/9184/2022.
