# Supplementary material for: Mass Mortality in Terns and Gulls Associated with Highly Pathogenic Avian Influenza Viruses in Caspian Sea, Kazakhstan
Source: Viruses. 2024 Oct 24;16(11):1661. doi: 10.3390/v16111661 (PMC11599136; doi:10.3390/v16111661)
Supplement: Supplementary file 1 [file viruses-16-01661-s001.zip › viruses-3128567-supplementary.pdf]

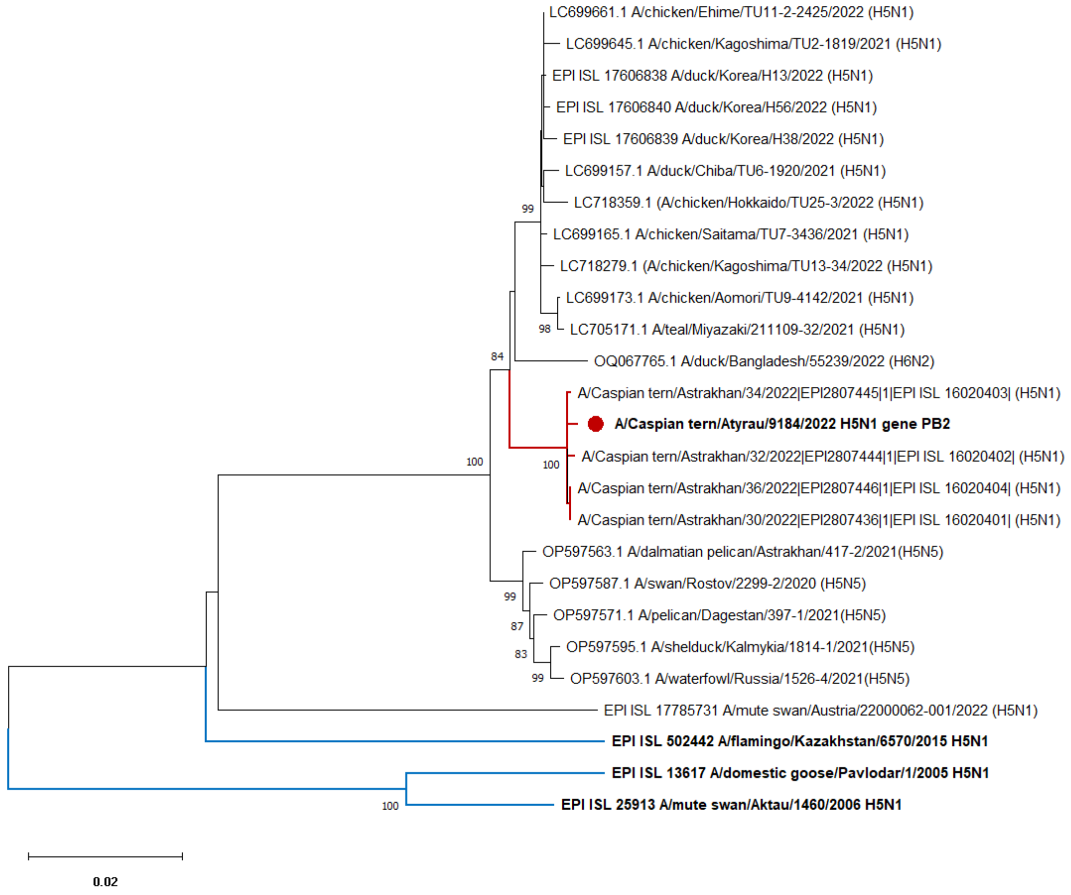

Figure S1. The phylogenetic tree of the full-length coding sequence of the *PB2* gene of the HPAIV H5N1 subtype A/Caspian tern/Atyrau/9184/2022 (marked with red dot) and other related viruses isolated from wild or domestic birds (GenBank and GISAID databases). The numbers at the nodes indicate the maximum likelihood bootstrap values of 500 replicates under the specified model. Only bootstrap values >70 are depicted. The bar represents a substitution rate of 0.02 nucleotides per site.

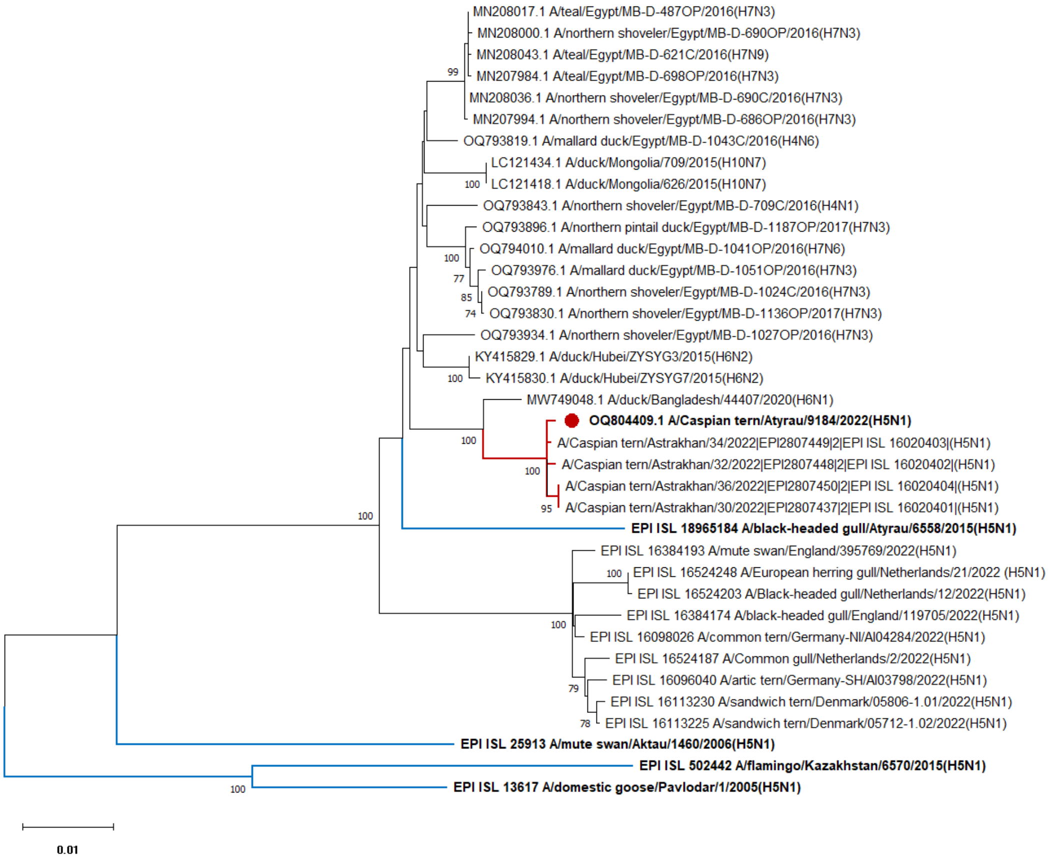

Figure S2. The phylogenetic tree of the full-length coding sequence of the *PB1* gene of the HPAIV H5N1 subtype A/Caspian tern/Atyrau/9184/2022 (marked with red dot) and other related viruses isolated from wild or domestic birds (GenBank and GISAID databases). The numbers at the nodes indicate the maximum likelihood bootstrap values of 500 replicates under the specified model. Only bootstrap values >70 are depicted. The bar represents a substitution rate of 0.01 nucleotides per site.

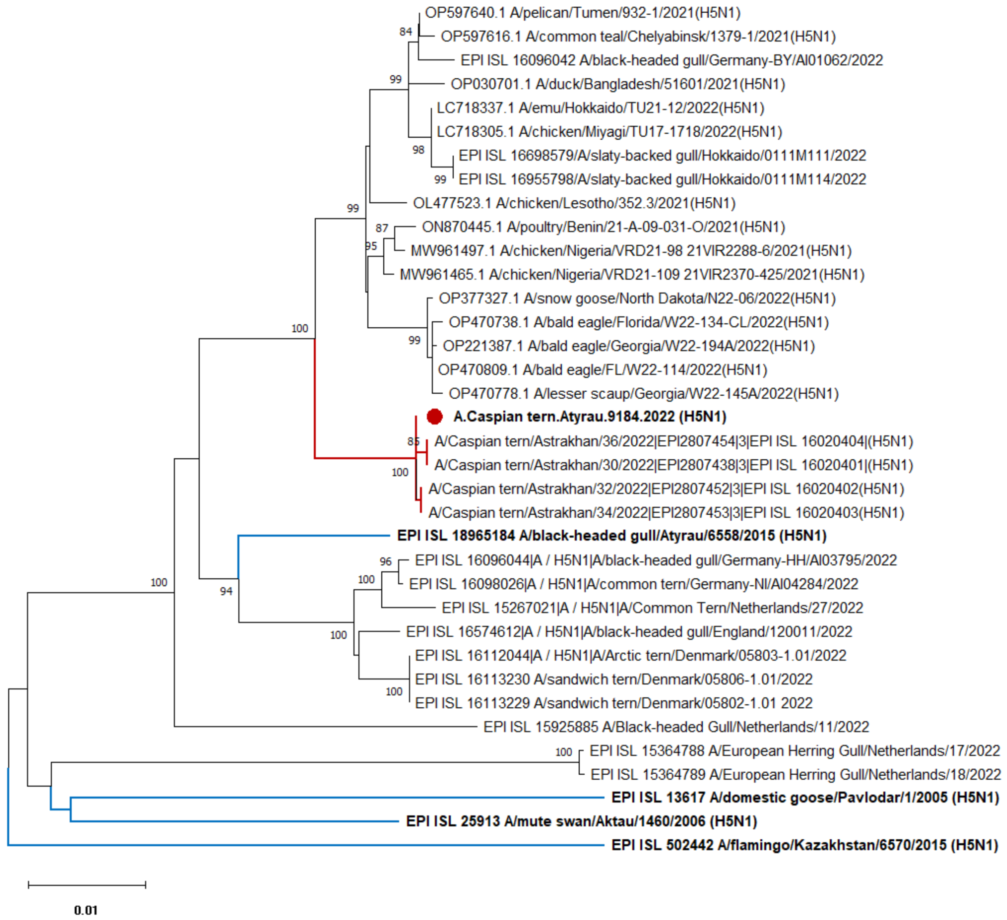

Figure S3. The phylogenetic tree of the full-length coding sequence of the *PA* gene of the HPAIV H5N1 subtype A/Caspian tern/Atyrau/9184/2022 (marked with red dot) and other related viruses isolated from wild or domestic birds (GenBank and GISAID databases). The numbers at the nodes indicate the maximum likelihood bootstrap values of 500 replicates under the specified model. Only bootstrap values >70 are depicted. The bar represents a substitution rate of 0.01 nucleotides per site.

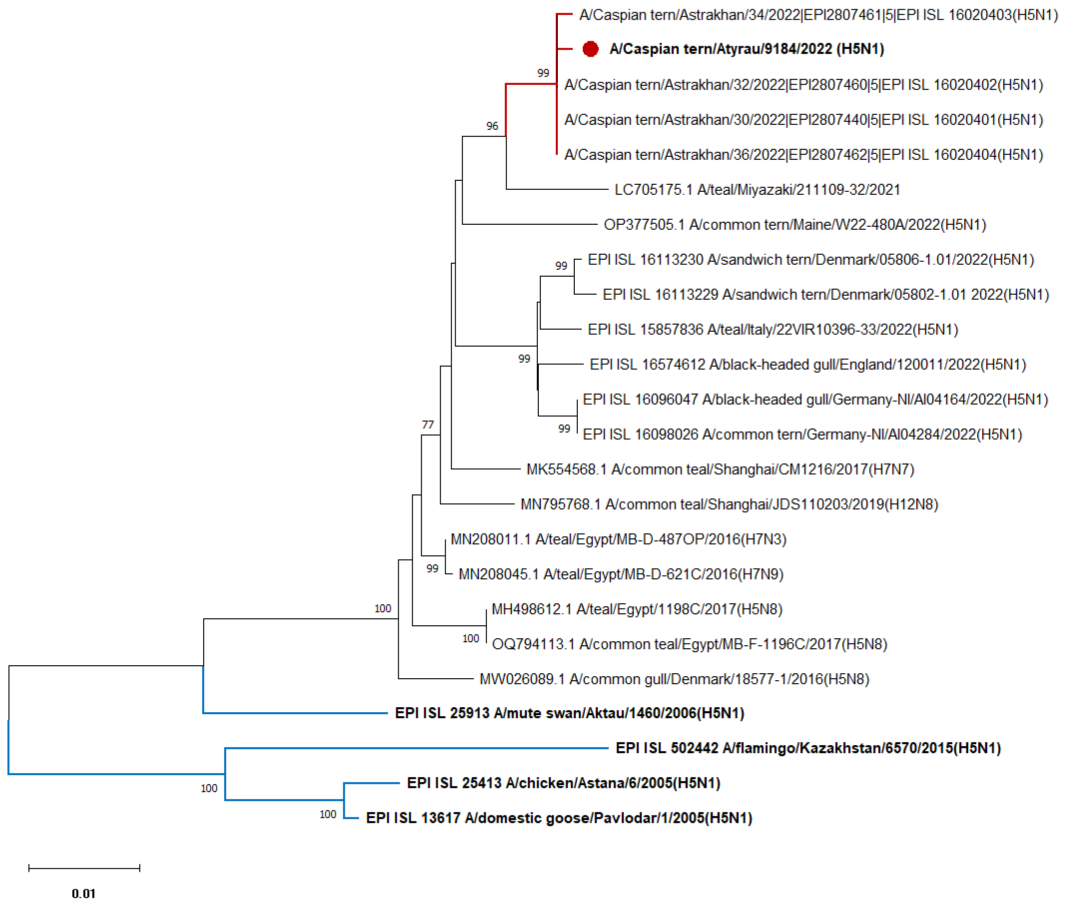

Figure S4. The phylogenetic tree of the full-length coding sequence of the *NP* gene of the HPAIV H5N1 subtype A/Caspian tern/Atyrau/9184/2022 (marked with red dot) and other related viruses isolated from wild or domestic birds (GenBank and GISAID databases). The numbers at the nodes indicate the maximum likelihood bootstrap values of 500 replicates under the specified model. Only bootstrap values >70 are depicted. The bar represents a substitution rate of 0.01 nucleotides per site.

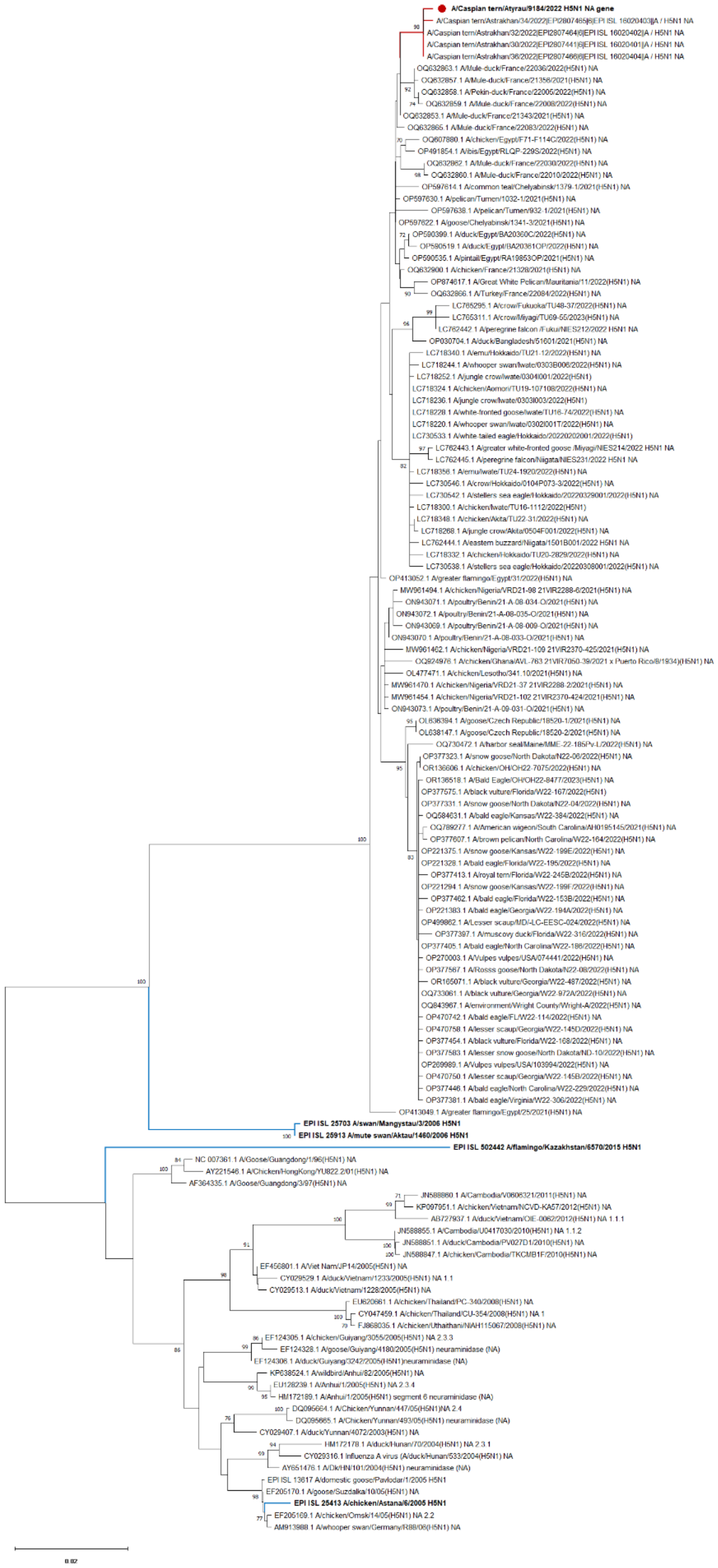

Figure S5. The phylogenetic tree of the full-length coding sequence of the *NA* gene of the HPAIV H5N1 subtype A/Caspian tern/Atyrau/9184/2022 (marked with red dot) and other related viruses isolated from wild or domestic birds (GenBank and GISAID databases). The numbers at the nodes indicate the maximum likelihood bootstrap values of 500 replicates under the specified model. Only bootstrap values >70 are depicted. The bar represents a substitution rate of 0.02 nucleotides per site.

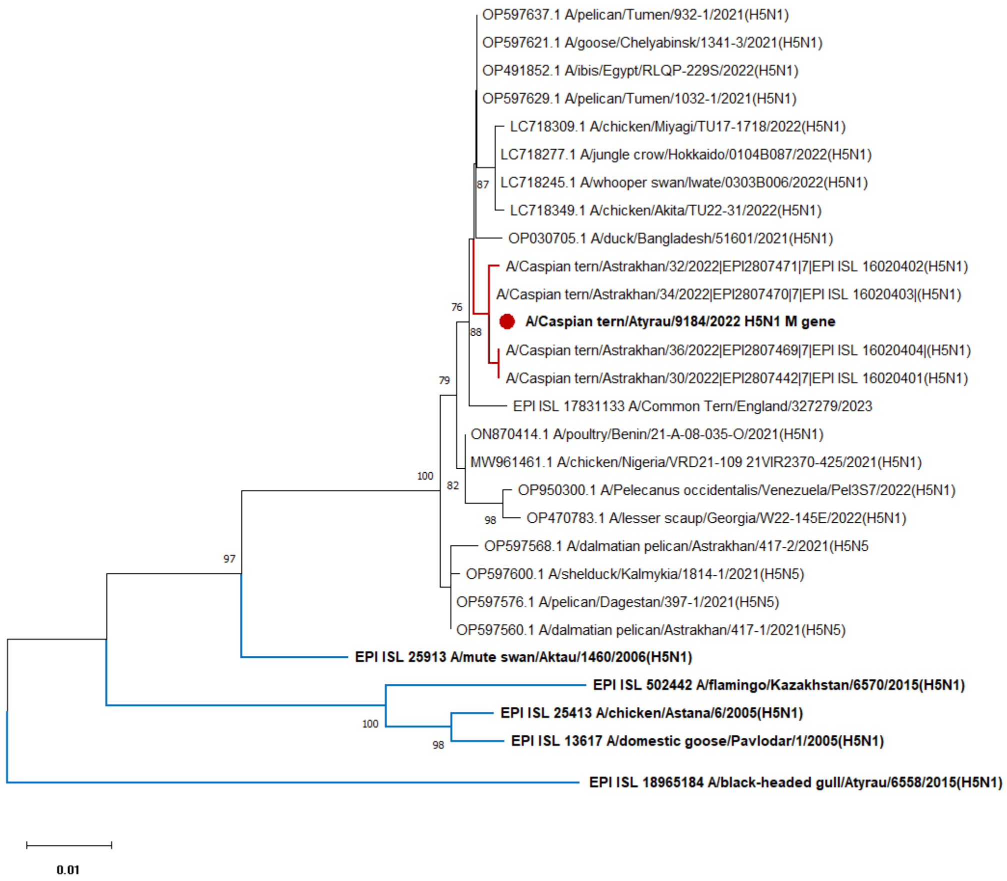

Figure S6. The phylogenetic tree of the full-length coding sequence of the *M* gene of the HPAIV H5N1 subtype A/Caspian tern/Atyrau/9184/2022 (marked with red dot) and other related viruses isolated from wild or domestic birds (GenBank and GISAID databases). The numbers at the nodes indicate the maximum likelihood bootstrap values of 500 replicates under the specified model. Only bootstrap values >70 are depicted. The bar represents a substitution rate of 0.01 nucleotides per site.

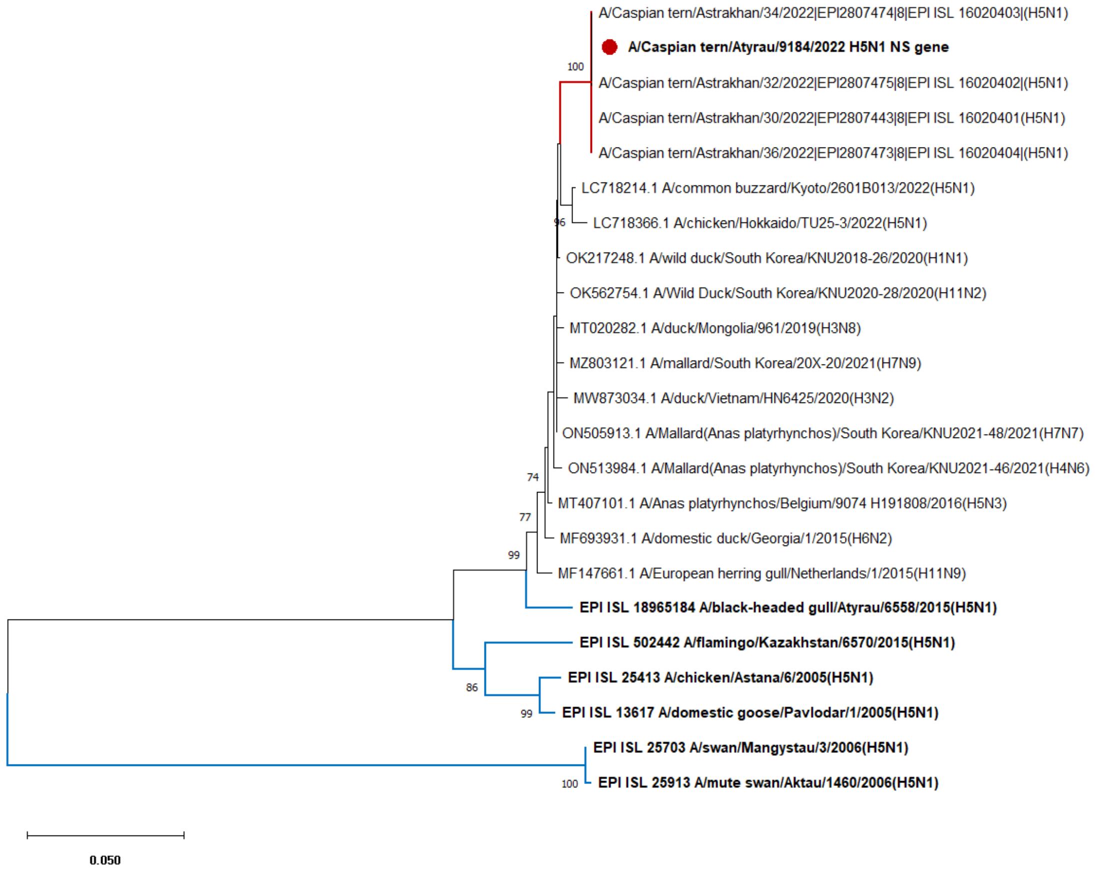

Figure S7. The phylogenetic tree of the full-length coding sequence of the *NS* gene of the HPAIV H5N1 subtype A/Caspian tern/Atyrau/9184/2022 (marked with red dot) and other related viruses isolated from wild or domestic birds (GenBank and GISAID databases). The numbers at the nodes indicate the maximum likelihood bootstrap values of 500 replicates under the specified model. Only bootstrap values >70 are depicted. The bar represents a substitution rate of 0.05 nucleotides per site.
